# Surfing uncertainty with screams: predictive processing, error dynamics and horror films

**DOI:** 10.1098/rstb.2022.0425

**Published:** 2024-01-29

**Authors:** Mark Miller, Ben White, Coltan Scrivner

**Affiliations:** ^1^ Centre for Consciousness and Contemplative Studies, Monash University, Clayton, Victoria 3800, Australia; ^2^ Department of Psychology, University of Toronto, Toronto, Ontario, Canada M5R 0A3; ^3^ School of Media, Arts and Humanities, University of Sussex, Brighton, BN1 9RH, UK; ^4^ Recreational Fear Lab, Aarhus University, 8000 Aarhus, Denmark

**Keywords:** predictive processing, horror, valence, emotion regulation

## Abstract

Despite tremendous efforts in psychology, neuroscience and media and cultural studies, it is still something of a mystery why humans are attracted to fictional content that is horrifying, disgusting or otherwise aversive. While the psychological benefits of horror films, stories, video games, etc. has recently been demonstrated empirically, current theories emphasizing the negative and positive consequences of this engagement often contradict one another. Here, we suggest the predictive processing framework may provide a unifying account of horror content engagement that provides clear and testable hypotheses, and explains why a ‘sweet spot’ of fear and fun exists in horror entertainment.

This article is part of the theme issue ‘Art, aesthetics and predictive processing: theoretical and empirical perspectives’.

## 1. Introduction

Despite tremendous efforts in the fields of psychology, neuroscience, media and cultural studies, it is still something of a mystery why humans are attracted to fictional content that is horrifying, disgusting or aversive. Some recent empirical research has identified clear psychological benefits in engaging in frightening play or frightening content (horror movies, horror video games, etc; e.g. [1]). However, it is puzzling that engagement and positive effects should arise from interactions with media specifically engineered to elicit negatively valenced affective states like fear or disgust. On the surface, these findings appear contradictory. In response to this, our goal in this paper is to provide an account of the cognitive and affective mechanisms at work beneath the surface of our engagements with frightening media.

Our account of the cognitive and affective mechanisms behind engagement with frightening media draws on a unifying theory of affect and cognition known as Predictive Processing (PP). According to PP, the brain's fundamental and sole imperative is to predict its own sensory states and to infer the causes of those states by minimizing the discrepancy between prediction and sensory input (i.e. prediction error). In this paper we argue that PP has the theoretical scope to explain how it is the case that engagement with media that reliably elicits negatively valenced states can lead to positive outcomes. We show how the affectively charged uncertainty associated with horror content serves as a potent learning signal, offering a controlled opportunity to occupy the limits of our predictive capabilities. We explain below how reward systems in the brain evolved to track and encourage our engagements with information at the edge of our own understanding (at the edge of informational chaos) where we find the most error-reducing opportunities, and how horror content co-opts these systems.

The structure of the paper will be as follows: in §2, we provide a quick primer on the PP framework, with a particular focus on how PP explains curiosity, play and exploration, and how these behaviors are inextricably linked to our affectivity and emotion. In §3, we identify the key features of horror content, and show how these features are especially potent in their capacity to push the predictive capabilities of the viewer. In §4, we show how PP elucidates the beneficial consequences of horror content, casting these benefits in terms of learning about the world, but also about ourselves and our capacity to control our thoughts and emotions. Finally, in our concluding section, we turn briefly to some of the negative outcomes that have been associated with horror content, drawing on existing PP literature on psychopathology.

## 2. Predictive processing, curiosity and play

According to PP the brain is understood to use a generative model (a model of the statistical regularities that underlay changes in the world) to make predictions about its own future sensory states. Through a process of belief updating and embodied action, the brain acts to minimize any discrepancy between its predictions and its actual sensory states—a quantity known as ‘prediction error’.^1^ So long as the organism keeps the error in its predictions to a minimum over time, either through updating its predictions or by acting on the world to make it better fit the prediction, the organism will typically succeed in achieving the valued outcomes it aims for in acting [2–7].

The generative model is hierarchical, with higher levels tracking longer timescales and more abstract features, while lower levels track faster, moment-to-moment causal regularities. Predictions cascade downwards while prediction errors flow upwards, driving learning and revised predictions [5,8].

At first glance we might think that this framework casts human agents as surprise avoidant, perhaps apt to seek the comfort of a dark room. This, however, is not typically the case (at least not for any prolonged amount of time). Despite its focus on uncertainty minimization, uncertainty itself has been shown to be intrinsically valuable, with agents being shown to seek uncertainty, find pleasure in its resolution, and to aim to occupy a band of uncertainty which lays at the edge of the agent's ability to cope. Known as ‘error dynamics’ this aspect of PP holds that the PP system is sensitive not just to the overall level of prediction error, but to the *rate* of error reduction through time, relative to expectation [9–12]. This rate of reduction through time can be visualized simply as a sloping line on a graph.

The rate of reduction relative to expectation has been directly linked to valenced bodily affective states. When we exceed expectations at reducing prediction error we feel good; when we underperform we experience negative feelings of frustration or despair (see also [13]). Recent evidence shows that humans are attracted to, and find pleasure in, relatively uncertain and volatile environments precisely because those environments afford excellent opportunities for error reduction at a better than expected rate—i.e. they offer optimal error dynamics that are essential for maximizing learning (see for example [10,14–17]). Learning is crucial to a system's ability to minimize uncertainty over time; even successful action policies are very often time restricted. For example, foraging in certain highly predictable areas for food might bring short or medium-term reward, but at some point the supply will be exhausted and new ground will have to be explored. Under PP these exploratory, or *epistemic* actions, are said to minimize expected prediction error on future outcomes [18]. In other words, for the predictive agent both familiarity (predictability) and a curiosity for novelty (unpredictability) drive adaptive behaviour. This is why we seem to be simultaneously attracted to both a comfort in familiarity and the surprising and unpredictable. Error minimizing creatures can then be characterized as *slope-chasers* who are motivated to seek out optimal opportunities to reduce prediction error, or uncertainty, in new and interesting ways [19–21].^2^

The last, and crucial, piece of this error dynamics picture is that in PP, reward learning systems play a central role in setting precision weighting on action policies. Precision weighting is a crucial mechanism within the PP architecture that estimates the importance and reliability of specific prediction errors, and modulates the impact those errors will have in revising beliefs and driving action. Errors with high precision are considered ‘newsworthy’ by the system, and will therefore drive further processing, while low precision renders errors relatively impotent within the system. Precision affords PP systems a high degree of contextual flexibility, both in what we attend to, and what kind of action policies are employed to achieve the desired state [4,22]. In other work, we have suggested precision is estimated and updated in real time in part based on error dynamics [11,23,24]. That has the implication that the success or failure of our actions in resolving error feeds back into the system to up- or downregulate precision on action policies going forward. Thus, error dynamics play a critical role in driving learning and keeping us behaviourally well adapted to the environment. For example, when we move to a new city we might take some time to dial into a new routine, learning about the most efficient routes to travel, the best places to get coffee, etc. Unexpected successes in this endeavour will increase precision on those policies, making it more probable that we repeat them and potentially forming those policies into habits along the way. When we fail to learn anything useful about our new home through a particular strategy (say, wandering around exploring in a certain direction), or we unexpectedly do worse than we have now come to expect at moving through the city for example, we might switch our approach to one we expect to be more efficient (catching a taxi instead of waiting for our regular train).

In short, the best slopes of error reduction (i.e. rewards) lay around the limits of an agent's embodied and psychological capabilities, at the ‘Goldilocks’ zone between too easy/boring and too difficult/unresolvable (see [25] or [26]). This attraction to the edge of our capabilities has been highlighted in experiments measuring looking times in human infants. It has been shown that infants prefer stimuli that are neither too simple nor too complex ([19]; cf. [27–29]; cf. [30], also see [31] for similar ideas). It is this drive to occupy areas of useful uncertainty that offer rich opportunities for learning that have been said to drive exploratory behaviour, underpin our inherent curiosity, and to lead us to engage in creative play [20]. We will argue that fictional horror can be attractive, in part, due to its ability to push us to that edge of our predictive capabilities. The better it is able to do this, the more engaging and rewarding it will be. This is a large part of our story, but it is not the whole story.

## 3. How horror content is engineered to be so potent

Films of all kinds can offer unique opportunities to slope-chasers like us. For prediction error minimizing agents, finding the good slopes is what it is all about, because good slopes are where learning takes place, which in turn allows us to better model the environment and thus ensure minimized prediction errors over the longer term. Horror films represent a special subclass of this attraction, one that can not only elicit strong affective responses, but also be especially useful for learning. The main point of this section is to highlight that these effects are not accidental; horror films are a particular kind of affective *technology*. They are designed carefully, using age-old tropes, cliches, and very specific kinds of sound and imagery, to elicit exactly the kinds of responses that they do.

Recent work suggests that we might enjoy horror films because they allow us to explore threatening situations, and so help us to transform our unpredictable reality into a more predictable one [32,33]. The monsters that are for us such a provocative source of fear and disgust are only the creative constructs of our imaginations, and appear only on the screens and pages of works we know to be fictional. However, under the PP framework, the boundary between perception and imagination is not always a very sharp one. Chris Frith has gone so far as to suggest that ‘our perceptions are fantasies that coincide with reality’ [34, p. 135], while Anil Seth has described perception as a ‘controlled hallucination’ [35].

Our perceptions originate in the prior expectations encoded in the generative model. The incoming sensory signal is used to correct the predictions of this model when discrepancies with the environment lead to prediction errors. Jakob Hohwy has suggested ‘conscious experience is like a fantasy or virtual reality constructed to keep the sensory input at bay. It is different from the conscious experience that is truly a fantasy or virtual reality, which we enjoy in mental imagery or dreaming, because such experiences are not intended to keep sensory input at bay’ [3, pp. 137–138]. Imagination here is understood as the process of simulating possible futures at higher layers of the generative model as part of the process of selecting action policies that minimize expected prediction error. This process of action selection thus requires taking into account possible sensory outcomes of future actions, and selecting the outcome that performs the best at keeping future prediction errors to a minimum [18]. Thus, perception, action and imagination form a ‘tangled skein’ because all of these processes work in the service of selecting action policies that minimize expected prediction error [36]. Generative models are inherently probabilistic, meaning that we actually perceive the world through models estimated to be more or less likely. In other words, as far as the predictive mind is concerned, films are not really untrue, as much as simply improbable. Yet, importantly, unlikely models can still be used for slope chasing, error resolvement and for learning.

Humans and their ancestors have been in competition with and hunted by large-bodied carnivores—mostly felids—for millions of years [37–39]. While the carnivores that preyed upon humans and other primates vary in many ways, they share some core ‘terrestrial carnivore’ features, such as large teeth, large claws and for the felids, stealthy ambush behaviour [37,38]. Those who were inefficient at detecting and dealing with ambush predators in particular would have suffered incredible fitness costs. Thus, it is reasonable to believe that humans have evolved some perceptual and behavioural response tendencies in response to high levels of predation (see e.g. [40–44]). Even young children, for example, demonstrated prepared social learning about dangerous animals [45]. Like other animals, humans and their ancestors have evolved in tandem with predators.

But what about monsters? While it is certainly the case that the monsters of horror films are fictional, they are nevertheless very deliberately composed of qualities and features that we have evolved to find salient [38,46,47]. Take werewolves, for example. Werewolves, much like real predators, hunt in the shadows, are large and formidable, possess sharp claws and teeth, and embody a threat of deadly violence. And it is not just werewolves: nearly all horror monsters and villains have attributes that mimic mammalian predators. Jason Voorhees, Freddy Krueger, Michael Myers and Leatherface are all ‘slasher*’* villains for a reason—their method of killing is associated with a sharp object. Behaviourally, nearly all horror villains engage in ambush and stealth behaviours, much like felid predators. Sharp appendages mimic the dangerous features of natural predators that we evolved long ago to attend to, and these dangerous features seem to lock onto deep, primal expectations about avoiding these sorts of homeostasis-jeopardizing signals in the wild [42,48]. They tap into phylogenetically old predator–prey relationship dynamics whereby carnivorous ambush predators capture and subdue prey using sharp, natural weaponry, namely, sharp teeth and claws. Sharp teeth and claws are universal features of mammalian carnivores and offer a recurrent ecological cue of danger for prey animals, including humans.

If horror was about the body count, all horror villains would carry automatic rifles. However, horror is about fear. A chainsaw is not a very effective method for killing a group of teenagers. It is heavy, loud and can run out of fuel. However, we have good reason to think that it strikes fear in us in part because its features (sharp saw teeth and loud ‘roar’) mimic features of mammalian predators (see [38,46,47]).

We have already shown that a ‘just-right’ amount of fear (i.e. resolvable error) is rewarding [49]. Chainsaws as a feature in horror (as opposed to, say, a gun) could potentially offer more interesting uncertainty to resolve (i.e. what it is and its role in hurting us). Loud, guttural and abrupt noises represent to humans, due to evolutionary and phenotypical prior expectations, highly reliable (precise) opportunities to gain life-sustaining information—information about the scene that helps us continue to enjoy the highly expected states associated with staying alive (see [50]). Humans are highly sensitive to finding relevant pathways for resolving current states of uncertainties, even if such pathways are purely fictional, counterfactual and even highly improbable. Moreover, uncertainties about threat hold primacy over other uncertainties due to the direct consequences of threats to survival and reproduction. In PP terms, threats like the ones depicted in horror films are threats to high-level expectations about homeostasis and self-organization. Because horror entertainment and other forms of recreational fear [49] are crafted to deal in these kinds of primal threats, they expertly co-opt reward systems designed to keep us learning about these important uncertainties.

As well as portraying monsters and villains that exemplify and accentuate the kinds of features that we have learned, over thousands of years, to fear, horror films are also carefully written to have plots and narrative arcs that keep us on the edge of our seats, making sure our attention is gripped to the most salient aspects. A full analysis of the techniques and intricacies of horror screenwriting are beyond the scope of this section, but it is worth taking a brief look at how uncertainty plays a key role. In many horror films, it is common for anticipation to play a central role in heightening states of arousal. For example, uncertainty around when the killer will strike, and who their next victim will be. A common horror technique is the ‘jump scare’, consisting in a palpable build up (using a range of visual, sound and narrative techniques), leading to an eventual sudden appearance or kill by the monster. The efficacy of the jump scare lies in the anticipation of an expected stimulus. Interestingly, research into gambling addiction has shown that what gamblers find most arousing (and what is most addictive) is the *anticipation* of a reward, rather than the reward itself [51–53]. What makes the jump scare even more effective, is that we are sometimes provided the build up, cueing our arousal and anticipation for a scare, only to find that the scare never comes. This intermittency of stimulus is extremely powerful. Indeed, in the case of gambling we know that a cue that signals a reward 50% of the time is most arousing, and experiments have shown that reward response decreases closer to a 100% pairing of cue and reward [54]. In a sense then, we can view the deliberate engineering of horror tropes as not dissimilar to the way in which games of chance have been engineered to be as arousing as possible to gamblers.

While the tropes outlined above—anticipation over the next victim, anticipation of potential jump scare—trade on our arousal in the face of intermittent stimulus, other horror movie tropes trade on our inherent attraction to familiarity. Indeed, to achieve the kind of slopes of prediction error minimization we are attracted to, we need a combination of the familiar and the surprising. The more predictable elements can be found in horror cliche; the ‘final girl’ trope from horror movies, for example, dictates that the last person standing at the end of the killer's rampage be a smart, capable woman who ultimately escapes or defeats the villain (think of Ellen Ripley in *Alien*, or Laurie Strode in *Halloween*). Ultimately though, the final girl's victory is nearly always temporary, as another cliche sees the monster never quite being confirmed dead, always poised for one last scare or a sequel. These tropes and cliches are so well-worn that movies like *Scream* made a point of making them explicit and playing on them to violate our expectations. Indeed, for veteran horror fans, some films deliberately violate expectations to provide the necessary uncertainty for those of us who require a little more challenge. *The Blair Witch Project*, for example, violated nearly all norms of horror, having no final girl, no resolution, and a painfully ambiguous threat. As endlessly playful and original as the genre can be, it remains the case that horror content generally, and movies in particular, exhibit their qualities not by accident but to deliberately elicit a set of affective responses in the viewer, and to grip their attention as powerfully as possible; this is their design.^3^

## 4. The benefits of engaging with horror

In this section, we bring together the previous two sections to bring out the beneficial features of our interactions with horror content. We describe how the very deliberately engineered features of horror interact with the cognitive machinery of the predictive agent in ways that drive learning and increase control.

### (a) Morbid curiosity and epistemic grip

One of the fundamentals to understanding PP and the power that a predictive mind has in keeping us alive and well attuned to the environment, is to understand that under PP, agents are understood to be closely coupled with their environments in the sense that the internal state of the system is partly defined by the external states of the environment and vice versa, mediated through active and sensory states. This close and reciprocal coupling of states means that we can view the environment and the agent as two parts of an integrated agent–environment system [11,55]. Environments though are rife with uncertainty and noise, and can potentially be volatile (meaning that the levels of uncertainty and noise can themselves be subject to rapid change). Thus, the embodied brain is always looking to maximize its epistemic grip on the world through precisely the kind of exploratory actions that drive learning, mentioned earlier. ‘Grip’ here simply means the capacity of the system to flexibly switch between action policies that reduce uncertainty consistently across short, medium, and long timescales [11]. These epistemic actions expressly forego achieving an immediate pragmatic end (like exploring for a new foraging site rather than continuing to exploit the existing, perhaps dwindling one), and instead take up policies that reduce uncertainty that shrouds a clear path to a future goal. Imagine, for example, once again navigating the new city. Getting to a desired goal might be a lot easier if we identify certain markers and way points. I might not know the way to the station from my current location, but I might know the way from a particular landmark and so head there first, *even though* this might increase my overall journey time and involve more physical exertion [56]. Notice here that predictive agents may sacrifice local prediction error gains (e.g. increased travel time; greater exertion) in order to achieve longer-term prediction error minimizing success. As we learn what an environment can offer, and begin to model the ways we can reduce prediction error by exploiting available resources, doing better than expected will *always* involve exploratory behaviors that allow us to keep learning and finding new resources. In short, predictive agents are driven to minimize their expected prediction errors over multiple scales of future outcomes, and this means always being tuned in to opportunities for learning.

Horror films can provide a useful source of information for learning about potentially volatile and highly newsworthy scenarios. In short, the claim is that fictional horror content provides a relatively low-stakes arena for learning about high-stakes scenarios. A similar argument has already been made regarding the widespread fascination, or morbid curiosity, with true-crime media [57] which is a phenomenon attested to by the prevalence and popularity of serial killer documentaries on streaming platforms like Netflix. Scrivner and Clasen [57] argue that morbid curiosity that leads us to engage with these kinds of shows is an epistemic drive: true crime offers a relatively low-risk strategy for learning what to look out for so that we can recognize and avoid dangerous individuals. In societies like ours, where the intent of violence and predatory behaviour is very often hidden from view, we naturally view these types of shows as providing potentially useful information about the kinds of clues and signs that might belie murderous intent in a stranger [58]. It is easy to see how the same logic applies in the case of horror fiction. In the previous section we highlighted how the fictional monsters and ghouls of horror turbo-charge the features of very real predators. Although the specific monster in a horror film may not exist, its embodied features and predatory behaviours closely track very real threats. Moreover, the actions—both successful and unsuccessful—that characters take (e.g. splitting up, going into the unlit basement, not closing the door behind you) provide learning opportunities (about how confident we should be in some set of actions in volatile situations) that generalize to potentially dangerous situations beyond the specific threat that is portrayed. In fact, fiction is *particularly* well-suited to showcasing critical teaching moments, especially when real-world examples are lacking [59].

It is even more striking that the kind of horror content we are driven to consume will often mirror contemporary events. In 2020, as the world was coming to terms with being in the grip of a pandemic, *Contagion*, a film about a deadly virus spreading across the world, shot up to become one of the top-ten most rented films, despite being released in 2011 [60]. The point here is not that *Contagion* is necessarily realistic or offers any true facts about *our* world. Rather, it is that films (and other forms of fiction) can in that sense be thought of as epistemic opportunities for exploring and learning about *possible* worlds, and about what opportunities those worlds might offer for optimal slopes of prediction error minimization. Cross-cultural evidence shows that humans of all places use fictional storytelling as a method of communicating factual information [61]. It might sound concerning to hear that adults learn and incorporate facts presented in fictional stories into their general real-world knowledge, even when such facts are false. However, there is evidence that this type of learning is useful [59]. For example, hunter–gatherers use storytelling to teach important social and ecological knowledge to others in their group [62,63]. Recently, Scrivner *et al*. [32] found that people who had seen more horror films reported increased psychological resilience during the early months of the Covid-19 pandemic. This suggests that frightening films in particular might also serve adaptive purposes by offering them a safe space to encounter and process (better predict) anxiety-inducing stimuli [32]. In short, *Contagion* served as a source of information directly related to the uncertainty the world was beginning to navigate.

In terms of facilitating an improved epistemic grip, the value of a film like *Contagion* is two-fold. First, by acting as a reliable information source, the film will have a general impact on the types of epistemic behaviour the system is likely to engage in. In other words, it will drive a shift in what we pay attention to and how we seek useful information in those volatile situations [64]. For example, *Contagion* might teach us that, in the event of a pandemic, it is worth paying attention to things like hospital admission rates and the resiliency of critical supply chains. Second, by engaging with what is perceived to be a reliable source of useful learning, the system will expect its own actions to be more effective in dealing with emerging prediction errors in those sorts of situations and environments, reducing some of the negatively valenced feelings that are associated with a system predicting its own inefficacy [23]. In short, through engagement with horror content, we first learn that this kind of content is indeed useful and the sort of thing that can provide good information, and we also increase our expectations about our own competence in the kinds of high-stakes scenarios depicted in horror.

### (b) Horror as an emotion regulation technology

Frightening films can serve as a learning tool about what sources of information in the environment might be most salient and which actions are most effective in dangerous scenarios that are possible in a world like ours. But this learning, and the increase in control and confidence that it brings, do not just apply to our ability to model what is *out there*. It also applies to our own cognitive and affective dynamics themselves. Indeed, in order to keep us within homeostatic bounds a system must minimize uncertainty about what is going on internally, within the body. We argue that the more we are exposed to media that elicit strong affective responses, the more evidence the system can gather about its own embodied reactivity to changes in uncertainty. In other words, horror content can provide a source of learning for updating our own self models.

Following work done by Lars Sandved-Smith *et al.* [65] on metacognition, awareness and attention in PP, we suggest that as the system becomes more aware of its own lower-level states (like affective responses and attention profiles), it also increases its ability to *regulate* those internal states. Roughly speaking, metacognition refers to thinking about thinking, but in PP terms it refers to a system's beliefs about its beliefs. Indeed, the fact that PP necessitates the system making predictions about predictions (i.e. estimating the precision on beliefs) has led to the framework being described as ‘quintessentially metacognitive’ [66]. In the context of horror, this would mean coming to better predict the kinds of psychological and affective responses that we are likely to have, and thereby gaining more control of the nature and intensity of those responses. We cast this as metacogntion, because we are describing how horror content can prompt us to consciously redeploy attentional resources over the predictive and affective dynamics that are unfolding as we engage with the medium.

**Figure 1.**
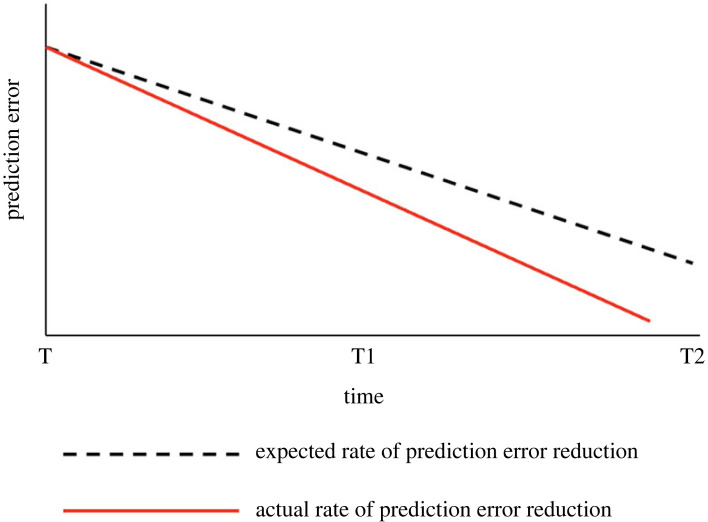
Actual rate of error reduction over time relative to the expected rate of error reduction over the same time period. The further below the expected rate the actual rate is, the more positively valenced affect the agent will experience. If the actual rate of error reduction moves above the line of expected rate, then the agent will experience a negatively valenced affective state.

To demonstrate the powerful role of metacognitive learning, Sandved-Smith *et al.* [65], draw on a distinction of a state being either transparent or opaque. Their work shows how through a process of increasing attentional awareness, states at lower levels can be made less transparent, and more opaque, thereby granting the system an increased degree of control [65]. This is because transparent states are not reflectively experienced as an object of thought (at least not in the present moment)—they are transparent in the sense that they are simply experienced as a part of the self, and because of this are generally highly motivating, driving selections of policies directly given our phenotype and habits [67].^4^ Error dynamics (described above, figure 1) are typically transparent, and drive action selection in ways that confirm the generative model relative to how one is doing at managing their cares and concerns. This means that the world as we experience it is experienced directly as emotionally charged; we experience things *through* our affective states, as merely a set of desires to be met or thwarted. As long as these processes are experienced transparently then they are taken to be a part of the self-model—these tunings are relative to the model we take ourselves to be. One way to think about transparency of error dynamics is in terms of a kind of ‘naive realism’ about our own affective dynamics, which is to say that transparency imbues those dynamics with the character of an unassailable force, or something inflicted upon us and entirely beyond our control.

Opacity, on the other hand, refers to the degree to which the state is ‘visible’ as an object of attention to our higher level attentional focus. There are different ways in which the internal dynamics of a system can become opaque. For example, one technique is mindfulness meditation, during which the system agentially turns its attention inward, to monitor the moment-to-moment fluctuations of those states. The opacification of this part of the precision machinery opens new opportunities for control [69]. Observing our valenced reactions allows us to develop new higher order policies about how precision (via valence) is being set on policies. In other words, instead of valenced signals adjusting precision on policies directly, and so automatically conditioning us to behave in certain ways, one can now learn to activate alternative policies depending on the usefulness of the valenced signals. For example, mindfulness has been shown to be highly effective in helping people to quit smoking cigarettes [70]. Similarly, it has been argued that dangerous sports—like mountain climbing or cave diving—allow agents to cultivate a certain type of self-awareness or self-mastery precisely because they force us to confront and control powerful and potentially unpleasant internal states [71].

We think horror movies can operate in a similar way, offering moviegoers a rare chance to learn about how their own nervous systems react in the face of specific kinds of stimuli, over which they lack any immediate control. When I decide to turn off the lights, and watch a particularly terrifying film alone, I set the scene to learn something important about *myself*. The experience is not just about what is ‘out there’ on the screen, it is also about what is going on inside me. For example, I might learn that my anxiety in a specific context tends to have a certain arc of activation—following a gradient from highly intense to less intense, and I might also learn that the arc of my anxiety response can be controlled if I remember to breathe, or keep in my mind certain rational mantras, e.g. ‘I know this can't really hurt me’. As we become more familiar with these natural rise and fall of prediction errors that accompany anxious situations, those slopes of error increase and reduction become more predictable, and so also less newsworthy—the prediction errors remain, but since they are now expected they no longer cry out to be immediately resolved.^5^

Learning about, and therefore rendering opaque, the internal affective dynamics of the predictive system changes our relationship to those dynamics in a non-trivial way, and can serve to ameliorate some of their more unpleasant elements. We can begin to see features of those dynamics—the bodily changes associated with anxiety, for example—not as an intrinsic and uncontrollable part of us, but as something apart from us, as information *about* us that we can, to a certain extent, choose to ignore or at least exercise a degree of control over. This allows us to be more tolerant to changes in uncertainty (it is taken to be less essential, less real); which in turn helps us to feel safe continually exploring new situations. Notice, though, that valence here does not go missing through this process of opacification but begins to be interpreted by the system as what it is: information that can be useful, but is not essential, in selecting policies.

### (c) Increased control over noisy interoceptive signals

One of the most unintuitive things about horror films is that, as a genre, horror seems to attract some fans who are high in trait anxiety and neuroticism (e.g. [6,7,72]). In other words, people prone to anxiety seem to be among the most likely to seek out movies with anxiety inducing plots. This seems puzzling; why would people who struggle with symptoms of anxiety deliberately seek out media designed to induce and heighten feelings of anxiety, fear and dread? In the previous two sections, we explained how horror content can serve as a learning technology, updating our models of both the external world (the features worth paying attention to), and of our own internal states (thus reducing the ungovernable force of our own emotional states). Here though, we explore how PP can offer an explanation of why horror can be appealing specifically to people who experience anxiety.

As we have seen, PP casts the brain as minimizing prediction errors associated with its own incoming sensory signals. By reducing uncertainty over the causes of its sensory states, the embodied brain increases its control of optimal action—it is more able to act on the world in ways that reduce prediction errors. But the brain is not only predicting exteroceptive sensory signals (signals from the environment), it is also predicting interoceptive signals (e.g. visceral, autonomic, hormonal signals, etc.). Interoceptive inference here is the process by which the brain maintains control of its own internal processes and metabolic functions during exchanges with the environment (the internal feeling of hunger prompting the actions of eating food, for instance). The process of maintaining homeostasis in the face of environmental change and volatility is known as allostasis [73–76]. Interoceptive inference (as central to allostasis) has, within the PP framework, been identified as centrally important to a range of psychological phenomena. For example, it has been suggested that interoceptive inference is central to our experiences of emotion [77–79], in underpinning symptoms of psychopathology [80] and to our sense of being a conscious ‘self’ [81,82].

Given the proposed centrality of interoceptive control to emotion and the self, it is not surprising that dysfunction in interoceptive inference is proposed to underlie a range of psychopathologies. For example, in the case of social anxiety disorder (SAD), Gerrans & Murray [83] propose an explanation of symptoms which focuses less on (mis)representation of external threat, and more on the ongoing process of maintaining healthy self-representations. As those authors note, ‘experience of internal visceral responses is critical for informing the organism of its current and anticipated cognitive-affective states’ [83]. According to the Predictive Processing account proposed by Gerrans and Murray, the symptoms associated with social anxiety result from uncertain, or ‘noisy’, interoceptive prediction errors pertaining to social encounters. Put another way, it is not uncertainty around things out there in the world (features of a particular social setting), but, rather, it is uncertainty about what state the agent is in as a result of being situated within the broader social setting. Just as in the case of exteroceptive signals, increased noise makes the brain's job of accurately inferring its own state more difficult, as it becomes harder to differentiate a reliable signal from the noise. Noisy environments are environments in which the embodied brain can struggle to exert control, and that rising degree of uncertainty is experienced as a negatively valenced affect, or stress, which in turn can have a detrimental effect on a range of physiological processes and systems [84].

More generally, anxiety and depression have both been associated with a reduced signal to noise ratio in interoceptive inference [85], or in other words, a reduction in certainty pertaining to information about the body's internal milieu. This reduction in certainty about the body in turn increases the associated negative affect that accompanies anticipating potential threats, failure, humiliation, and so on, as, if one cannot be sure about one's own internal states one also cannot be sure how to act appropriately and with confidence in the world. What is important for our purposes here, is that symptoms of anxiety arise through a perceived lack of control over interoceptive inference. This is perhaps most starkly demonstrated in empirical work that shows that a subject's level of interoceptive accuracy (their ability to correctly detect their own heart rhythms, for example) may be a factor in the experience of symptoms of anxiety and that training to improve heartbeat detection, for instance, correlates with a reduction in anxiety symptoms [86]. All of this demonstrates that there is a clear theoretical and empirical grounding for interpreting the engagement with horror by people prone to anxiety as a seeking for control.

It is worth emphasizing the point that uncertainty associated with noisy interoceptive (and exteroceptive) signals is something that predictive systems like us will go to great lengths to avoid. The perceived lack of control and, therefore, difficulty to act effectively directly contravenes the system's imperative to maximize its model evidence. This must be rectified, and so is experienced by the agent as feelings of frustration, anxiety and stress—the strong sense that we must make changes to do better. Being in this highly uncertain and stressed state is profoundly unhealthy, eventually exhausting and dysregulating hormonal and metabolic resources [76,87]. Given this potentially huge cost, it is hardly surprising that the predictive system will sometimes take drastic action—even adopting highly dysfunctional patterns of activity—to exert some sense of control over the self and the environment [76,84].

If a core problem with anxiety is a loss of interoceptive access and control, then one solution may be to create a highly controllable and sufficiently loud (precise) interoceptive experience. Laura Barca and Giovanni Pezzulo [88] have recently suggested food restriction in anorexia nervosa (AN) may serve just this purpose. Barca and Pezzulo argue for a novel computational account of AN, based in the mechanisms of PP. Their view presents empirical evidence that supports the view that anorexia (and other eating disorders) is fundamentally rooted in a patient's need to control uncertainty associated with interoceptive states. They write that ‘while the sources of uncertainty are potentially plentiful, in the case of AN (anorexia nervosa) the most relevant ones might be specifically related to bodily events; which in turn may have cascading effects across multiple domains (emotional, social, and related to the self)' [88, p. 428]. According to Barca and Pezzulo, the extreme restrictions on eating that characterize AN can be viewed as actions directed toward amplifying interoceptive hunger signals in a highly controllable and predictable way. The amplification of these signals increases their precision and thus reduces their noise, resulting in increased certainty about the body and an increase in the agent's sense of control over their internal states. This increased sense of control can be extremely difficult to relinquish.

While the behaviors and beliefs that characterize anorexia nervosa are disturbing and a direct threat to the health of the individual, and engagement with horror content is none of these things, it is nevertheless clear that the case of anorexia provides a clear context for understanding how horror content might also provide temporary relief for people who suffer from a generalized anxiety disorder. Horror movies also offer a highly controllable and predictable way to amplify certain interoceptive signals associated with fear: perspiration, increased heart rate, etc. and by doing so elucidating a clear and reliable covariance between an external cue (horror film) and an internal state (fear). This mirrors the clear covariance established in anorexia: the external cue (I am restricting my eating) and the internal state (I am hungry). This general idea is not without precedent; Scrivner and Christensen [89] have suggested that engaging with horror may help to provide a sense of control over anxiety symptoms in the short term and build resilience in the long term. Horror content, like restricted eating, simply provides a source of certainty about, and a sense of control over, our interoceptive system. By actively choosing to watch a horror movie one is able to, for a short time, feel as though their anxiety has a clear and controllable source—the horrible content on the screen.

While we might intuitively think that horror forms are relatively passive, and so lack the opportunity for us to exert any real control, this is not the case. There are lots of ways that we actively adjust the error profiles that we encounter during a movie, helping ourselves to locate and enjoy the best slopes on tap. For example, we choose how scary we think the film is (e.g. based on reviews) and what topics it covers (e.g. monster, contagion, etc.); we can actively control the sensory stream, and so our embodied responses to that stream, by peeking between our fingers or covering our eyes (see [90], for example); we can turn down the lights, which makes our vision—a primary means of gaining a good predictive grip—less precise which in turn reduces our precision (or confidence) on our own abilities to reduce error. This in turn makes the system more sensitive to novel errors, and so more susceptible to being interoceptively affected by the horror movie. It is also worth noting that in PP, there is a subtle distinction to be made between uncertainty that is expected (predicted) and uncertainty that is unexpected (generating prediction errors). Noise over interoceptive signals is almost always unexpected—as a fundamental operating principle, the brain expects to have clear access to what is happening inside the body, and any violation of this is newsworthy. Conversely, while horror movies offer up uncertainty (who will die first, who is the killer, where is the monster hiding, etc.), that uncertainty is very much expected, in the sense that we know we are about to watch a horror movie and know what that normally entails. In other words, the connection between our engagement with horror content and a specific set of interoceptive signals is a very predictable—and controllable—one.

In §3 we described how part of the engineering of horror content includes some very well-worn and predictable tropes and cliches, and it is these that help add to the increased sense of control over our internal states. For example, many experienced horror fans will expect a jump scare when the protagonist closes a medicine cabinet, exposing the monster or killer in the mirror's reflection. Other actions that characters take in horror movies can also signal impending threat. When a group of characters split up or go down into a dark basement, the audience expects a threat to be looming. Finally, the music that is common in horror movies can signal to the audience when a jump scare is imminent. Experienced horror movie-goers quickly learn which type of music indicates a threat. However, even novice horror fans can pick up on music that signals threat because the music in horror movies acoustically mimics screams, which evolved to signal threat [91]. Interestingly, terrifying music is also rhythmically unpredictable, which may prime our minds to pay attention and learn [92]. The upshot is, if we know a jump scare is coming, and we know it will elicit an embodied response, then that knowledge constitutes exactly the sort of increase in the agent's sense of control and subsequent reduction in interoceptive noise.

## 5. Concluding remarks, the potential dark side of horror

We have highlighted, using the computational framework of Predictive Processing, how horror films can, often in surprising ways, act as a powerful source of learning and control, attenuating feelings of anxiety and stress. In doing so, we showed that the apparent paradox of horror is nothing of the sort; even though horror movies are designed specifically to elicit affective responses we might generally think of as unpleasant, there is a real value in this that comes in many forms.^6^ However, it is worth noting in conclusion that this does not mean that our engagement with frightening media can never take a more pathological turn. Morbid curiosity can be driven by expectations about horrible outcomes, rather than by curiosity alone (see [96]).

In PP terms, saliency can be driven either by expectations that there are some meaningful and reducible errors to be resolved by curiously pursuing explorative actions, or by actively sampling the environment in well worn ways that confirm already highly precise expectations [97]. Recently, PP has provided a number of computational accounts of various psychopathologies including major depression [23,98]. A common symptom of major depression is learned helplessness, a condition in which the subject increasingly comes to believe in their own inefficiency in terms of being able to do anything to alleviate their symptoms [99]. The subject essentially installs a high-level prior belief that no matter what action they take, their situation will not improve. According to these PP accounts, learned helplessness arises from prolonged dyshomeostasis or high allostatic load [100]—the result of consistently having to deal with a highly volatile or uncertain environment for long periods of time. As mentioned earlier, the physiological costs of operating beyond homeostatic set points in this way can be significant. The persistent negative affect (arising from doing worse than expected at reducing error) eventually has the consequence that the person assigns low precision to all of their action policies. That is, there is a global loss of confidence that any action they will take will succeed in helping them adapt to their environment. A powerful feedback loop emerges here: the prior belief that one cannot act in ways that will reduce prediction error leads the organism to begin sampling the environment for evidence of this inability, which in turn confirms and supports the hopeless belief.

Some forms of horrifying content may then be attractive not only because they offer some valuable learning signal about the self or the world, but also because they are seen as an opportunity for us to confirm a set of prior expectations about the way the world is. Given what we have argued above about horror content confirming our expectations about the world, we might then expect people who have undergone traumatic experiences to be more likely to seek out horrifying content. While it might seem counterintuitive, there is some evidence that supports this. For example, it has been shown that peacekeepers deployed to Bosnia are more likely to have seen a TV drama about war [101]; people with PTSD are often attracted to media that reminds them of their trauma [102]; people who have suffered extreme violence are more likely to seek out violent videos online [103]; university students who lived near a classmate who was murdered were more likely to purchase tickets to a thriller movie at their local theatre [104].

Moreover, Mexico, which has 7 of the 10 cities with the highest murder rates in the world [105], is also the most horror-loving country in the world [106]. And of course the fact that the two most successful years for horror were 2020 and 2021 during the Covid-19 crisis, which were two of the scariest years for many people around the world [107]. Living in an extremely dangerous environment, such as a war zone is likely to minimize the time we spend watching movies or playing games. However, it seems that given the opportunity, those that have experienced dangerous situations *are* drawn to engage with this kind of media. Future research would need to examine if this association is linear or perhaps curvilinear. For example, if engaging in frightening media is subconsciously motivated by learning about threats [57], then being exposed to real danger over a long period of time may obviate the need to learn about it from media. Curiosity generally follows an inverted-U shape association with knowledge, such that too much knowledge about a topic decreases curiosity [27,108]. It is likely that curiosity about threats follows this same pattern.

One potential danger here is that if these prior expectations about the world are particularly negative, then the predictive system can enter into a vicious feedback loop, wherein it continues to engage with horrifying content to reconfirm its beliefs that the world is a horrible and threatening place—which in turn leads to a stronger attraction to horror content. Given the accounts of depression outlined in the previous paragraph, and this tight relationship between negative expectations and evidence gathering, we have a way into understanding why it is that repeated, compulsive engagement with horrifying material could result in pathological outcomes. A complete exploration of this pathway lies beyond the scope of this paper, but future work should pursue this line, especially today when the internet offers such a depth of potentially disturbing, graphic, real-world content.

## Data Availability

This article has no additional data.

## Endnotes

1 Throughout this paper we will refer to both ‘prediction error’ and ‘uncertainty’. We use these terms interchangeably, although strictly speaking ‘prediction error’ is a technical term referring to a sub-personal quantity computed by the cognitive system, while ‘uncertainty’ is a more general term also used to refer to the environment. E.g. environments can be rife with uncertainty (e.g. exploring a new city), and encountering and resolving that uncertainty necessarily entails the system minimizing prediction errors.

2 Notice that error dynamics are always computed relative to expectations about rates of error resolution in a specific context. That means that we are attracted specifically to spaces where we both have a good predictive grip (i.e. our models, skills and the environmental dynamics align in ways that allow us to know what is happening broadly over time), *and* where there are chances for improvements given this good predictive grip. Enjoying good error dynamics slopes necessitates the system delicately balancing the predictable and habitual, with the less predictable and more challenging opportunities in the environment.

3 Notice here that part of what slope chasers like us are doing when engaging with horror media is not only reducing uncertainty about potential real-world scenarios, but also tracking and enjoying the uncertainty and its resolution in the context of the film's narrative itself—part of what we are attracted to is working out the twists and turns of the specific narrative. However, this is not at all limited to the horror genre specifically, and we think gestures toward a broader theory about why predictive agents are drawn to fictional works generally. Thanks to a reviewer for highlighting this point.

4 For a specifically PP application of these ideas see [68].

5 See [10] for a full account of this phenomenon.

6 One might wonder why given this account of the attractiveness of horror content horror as a genre is not much more popular than it is (sitting at about 8.5% of market share for movies). While horror provides an excellent place to find, and be thrilled by, manageable prediction errors, those errors are related to sensations and topics that can be highly aversive, and so interact strongly with various personality traits like disgust sensitivity, sensation-seeking, morbid curiosity, etc. (for some research on the relationship between these personality traits and horror see [72,93–95]). This is all to say, even though entertainment can offer the kinds of slopes of error minimisation we are all drawn to, the specific tropes and aesthetics of those genres still matter. This is also why Romance is a relatively unpopular genre, despite all of us being members of a species that shares an evolutionary imperative to mate and reproduce. Finally, it is also worth noting that many horror movies are R rated, meaning that they fail to capture the family box office market and preclude many young fans who would otherwise be happily paying customers.
